# From proteomics to colloidal gold tests for urinary thrombomodulin: a prospective cohort study on accurate sepsis screening

**DOI:** 10.3389/fcimb.2026.1779950

**Published:** 2026-03-09

**Authors:** Qi Chen, Junjie Zeng, Lincui Zhong, Ziwei Jiang, Longping He, Qingwei Lin, Qingbo Zeng, Jie Liu, Huancai Yin, Jingchun Song

**Affiliations:** 1 Department of Critical Care Medicine, Changcheng Hospital Affiliated to Nanchang University, Nanchang, China; 2 Department of Critical Care Medicine, The 908th hospital of Joint Logistic Support Force, Nanchang, China; 3 Intensive Care Unit, Nanchang Hongdu Traditional Chinese Medicine Hospital, Nanchang, China; 4 Laboratory of Biomedical Testing Technology, Suzhou Institute of Biomedical Engineering and Technology of the Chinese Academy of Sciences, Suzhou, China

**Keywords:** colloidal gold tests, machine learning, proteomics, sepsis, septic shock, thrombomodulin

## Abstract

**Background:**

To develop a new non-invasive screening method for sepsis by detecting urine samples.

**Methods:**

A prospective study was conducted to collect urine samples from a cohort of 22 individuals diagnosed with sepsis and admitted to the Intensive Care Unit (ICU) of a university-affiliated teaching hospital in China. Utilizing proteomic and bioinformatics analyses, we sought to identify potential biomarkers indicative of sepsis. These biomarkers were subsequently validated using serum and urine samples from 31 patients with septic shock, 83 patients with sepsis, and 50 healthy controls. Receiver operating characteristic (ROC) curves were employed to determine the optimal cutoff values for these biomarkers. Based on the diagnostic thresholds derived from ROC analysis, colloidal gold test strips were developed and applied to screen a cohort of 92 ICU patients. The diagnostic accuracy of these test strips was rigorously assessed by comparing their results with those from immunofluorescence assays.

**Results:**

Data-independent acquisition (DIA) proteomics analysis of urine samples identified 2,846 proteins, with stringent filtration criteria (fold change > 2 or < 0.5, P-value < 0.05) yielding 178 differentially expressed proteins (DEPs). Kyoto Encyclopedia of Genes and Genomes (KEGG) pathway enrichment analysis revealed significant enrichment of DEPs in pathways associated with “cell adhesion molecules,” “lysosomes,” and metabolic processes. The Boruta algorithm, integrating Random Forest and Support Vector Machine (SVM) analysis, identified urinary thrombomodulin (TM) as a key candidate molecule. Immunofluorescence analysis for validation trial showed rising trend in blood TM levels across disease severities: 7.55 (6.58-8.72) TU/mL in healthy controls, 10.08 (8.00-14.15) TU/mL in general sepsis, and 12.30 (7.54-18.68) TU/mL in septic shock. Conversely, urinary TM levels decreased: 23.65 (18.08-31.06) TU/mL, 17.70 (13.80-28.80) TU/mL, and 5.84 (4.00-11.59) TU/mL, respectively. At a urinary TM threshold of 15.46 TU/mL, the ROC AUC for sepsis diagnosis is 0.72, with 57% sensitivity and 88% specificity (P<0.05), showing no significant difference comparable to blood TM (P>0.05). For septic shock diagnosis and 28-day mortality prediction, a urinary TM threshold of 11.85 TU/mL yields an ROC AUC of 0.92, with 93% sensitivity and 81% specificity, outperforming blood TM (P<0.05). A urinary TM colloidal gold test strip, which turns red at TM levels above 15.46 TU/mL, was developed and validated on urine samples from 43 sepsis and 49 non-sepsis patients, achieving 86.1% sensitivity, 77.6% specificity and an overall accuracy of 81.5% for sepsis diagnosis. The Kappa test validated the concordance of the colloidal gold strip test with Sepsis 3.0 diagnostic criteria, while the McNemar test indicated no significant difference in sepsis diagnosis efficacy between the strip test and chemiluminescent immunofluorescence (p=0.228).

**Conclusions:**

The utilization of urine test strips for the detection of TM offers a precise, convenient, and practical method for the screening of sepsis.

## 1 Introduction

Sepsis, a critical illness characterized by life-threatening organ dysfunction due to a dysregulated immune response to infection, accounts for approximately 49 million annual cases and 11 million fatalities, representing 19.7% of all global deaths (Singer et al., 2016; Rudd et al., 2020). Septic shock, marked by persistent hypotension despite fluid resuscitation, is associated with severe circulatory compromise, tissue hypoxia, metabolic derangements, and multi-organ dysfunction, with mortality rates exceeding 60% (Carlos Sanchez et al., 2023). Timely recognition of sepsis and prompt therapeutic intervention are vital for reducing mortality (Martín-Fernández et al., 2021). Procalcitonin (PCT) and C-reactive protein (CRP) are widely accepted as sepsis biomarkers, yet inconsistencies limit their diagnostic utility (Mierzchała-Pasierb and Lipińska-Gediga, 2019; Tujula et al., 2020). Serial blood sampling and longitudinal assessment of plasma concentrations are crucial for diagnostic precision (Long and Gottlieb, 2025). However, in the context of scarce blood resources, the risk of iatrogenic anemia resulting from frequent venous blood sampling and the concomitant risk of bloodstream infections present an escalating challenge in the management of sepsis (Jandu et al., 2019; Holland et al., 2020). This underscores the urgent need for non-invasive, dynamic, and accurate diagnostic tools for sepsis.

Data-independent acquisition (DIA) proteomics, known for its comprehensive quantitative proteomic profiling with high quantitative accuracy and reproducibility, is instrumental in elucidating disease mechanisms, identifying early diagnostic biomarkers, and targeting therapeutic interventions (Demichev et al., 2022). Plasma, a complex biofluid with a vast concentration disparity among proteins, is dominated by high-abundance proteins like albumin and immunoglobulin G (IgG), which can obscure low-abundance proteins (Fliser et al., 2007). Urine-based proteomics offers advantages such as stable protein composition, ease of collection, non-invasiveness, and continuous sample availability (Issaq et al., 2007). Urinary biomarkers like interleukin-10 (IL-10), neutrophil gelatinase-associated lipocalin (NGAL), and TIMP-2 have shown diagnostic potential in sepsis, but their clinical application is often impeded by high detection costs, complex methodologies, and suboptimal accuracy (Amin et al., 2024; Palmowski et al., 2024; Zhao et al., 2024). This study aims to leverage urinary DIA proteomics to identify novel biomarkers and develop a simple, user-friendly, and accurate sepsis screening tool using colloidal gold detection technology.

## 2 Methods

### 2.1 Study design and participants

From January to April 2024, we prospectively collected urine samples from 11 patients with sepsis and 11 with septic shock at the Intensive Care Unit (ICU) of Changcheng Hospital, affiliated with Nanchang University, to identify potential biomarkers through DIA-based proteomic and bioinformatics analyses. From May to October 2024, we obtained blood and urine samples from 31 patients with septic shock, 83 patients with sepsis, and 50 healthy controls to evaluate the diagnostic performance of blood and urine TM. From December 2024 to May 2025, we enrolled 43 patients with sepsis and 49 non-sepsis patients in the ICU to compare the diagnostic accuracy of urine TM assays with immunofluorescence and our novel colloidal gold assays for sepsis diagnosis.

**Figure 1:**
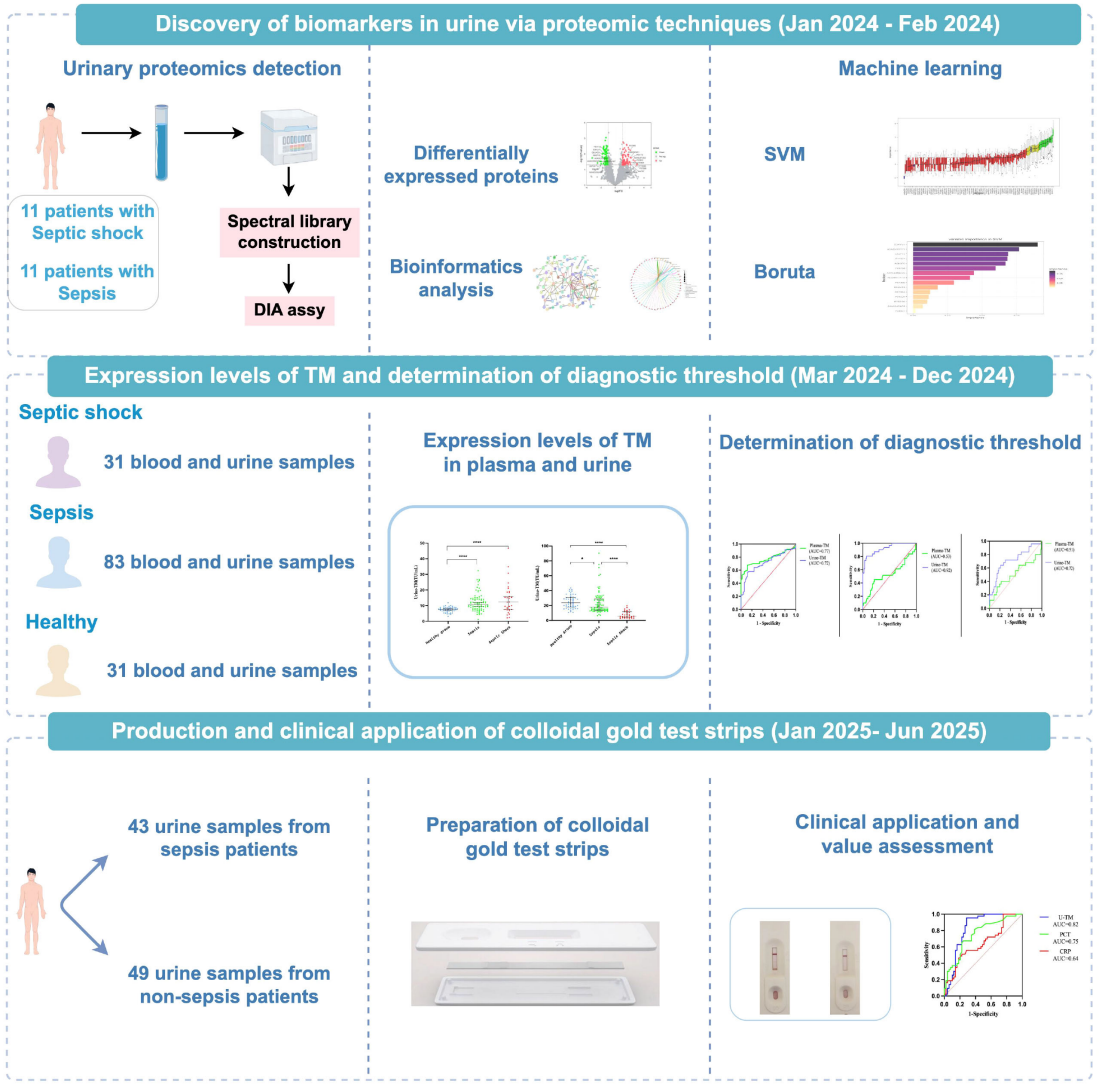
Experimental procedure.

Eligible patients met the following criteria: (1) age ≥18 years; (2) patients diagnosed with sepsis according to the 2016 Sepsis 3.0 criteria jointly published by the Society of Critical Care Medicine (SCCM) and the European Society of Intensive Care Medicine (ESICM), which define sepsis as a SOFA (Sequential Organ Failure Assessment) score ≥2 and evidence of infection (Singer et al., 2016). Exclude shock in which non-infectious factors are the primary cause of onset, and septic patients were diagnosed with septic shock if they required vasoactive agents to maintain a mean arterial pressure (MAP) ≥65 mmHg and had a lactate level >2 mmol/L, despite adequate fluid resuscitation (Singer et al., 2016). The exclusion criteria were: (1) anuric patients; (2) patients undergoing continuous renal replacement therapy; (3) chronic kidney disease; (4) patients who had undergone ureteral irrigation; (5) immunocompromised patients; (6) pregnant or lactating women; (7) individuals who refused to participate. The study was conducted in accordance with the ethical guidelines outlined in the Declaration of Helsinki and approved by the hospital’s ethics committee (approval number 908YYLL2024044). All family members of the patients have signed informed consent forms. The experimental protocol is depicted in **Figure 1**.

### 2.2 Blood sample testing methods

Within two hours of intensive care unit (ICU) admission for septic patients, and concurrently for healthy controls, corresponding blood samples were procured for a spectrum of diagnostic assays. For complete blood count (CBC) analysis, 2 mL of blood was extracted from septic patients utilizing EDTA anticoagulant tubes. The CBC tests were executed using a BC-6900 automated hematology analyzer (Mindray Bio-Medical Electronics Co., Ltd., Shenzhen, China), with parameters measured including white blood cell (WBC) count, absolute neutrophil count (ANC), absolute lymphocyte count (ALC), red blood cell (RBC) count, hemoglobin concentration, and platelet count.

For biochemical assays, 3 mL of peripheral venous blood was collected from both septic patients and healthy controls using serum separator tubes. These assays were performed with a BS-2000 automated biochemical analyzer (Mindray Bio-Medical Electronics Co., Ltd., Shenzhen, China), with parameters evaluated encompassing CRP, alanine aminotransferase (ALT), aspartate aminotransferase (AST), total bilirubin (TBIL), total protein, albumin, and creatinine.

Routine coagulation tests were conducted on 2 mL of peripheral venous blood samples from both septic patients and healthy controls, collected using citrate anticoagulant tubes with a citrate-to-blood ratio of 1:9. The tests were executed with an ACL TOP700 automated coagulation analyzer (Werfen, USA), assessing parameters such as prothrombin time (PT), international normalized ratio (INR), activated partial thromboplastin time (APTT), thrombin time (TT), fibrinogen, D-dimer, and antithrombin.

Immunofluorescence assays were performed on 3 mL of peripheral venous blood samples from both septic patients and healthy controls, collected using dry tubes. These assays were conducted with a UPT-3A up-converting phosphor immunoassay analyzer (Beijing Hotgen Biotech Co., Ltd., Beijing, China), with biomarkers assessed including N-terminal pro-brain natriuretic peptide (NT-proBNP) and PCT.

For blood gas analysis, arterial blood gas analysis was performed on septic patients using an ABL90FLEX blood gas analyzer (Radiometer Medical ApS, Denmark), with documentation of blood lactate levels within two hours post-admission.

### 2.3 Disease severity assessment

Acute Physiology and Chronic Health Evaluation II (APACHE II) and Sequential Organ Failure Assessment (SOFA) scores were calculated for septic patients. The incidence of Acute Kidney Injury (AKI) and the 28-day mortality rate were recorded. AKI was defined according to the Kidney Disease: Improving Global Outcomes (KDIGO) guidelines (Stevens et al., 2013), with criteria including: a serum creatinine (Cr) increase of ≥0.3 mg/dL (≥26.5 μmol/L) within 48 hours; a Cr elevation of ≥1.5-fold from baseline within the previous 7 days; and urine output <0.5 mL/kg/h for ≥6 hours.

### 2.4 Mass spectrometry experiments

#### 2.4.1 Protein extraction and peptide digestion

Urine samples were first centrifuged at 3,000 g for 10 min at 4 °C to remove cell debris. The resulting supernatant was concentrated and desalted using a 10 kDa molecular weight cut-off (MWCO) centrifugal filter (Millipore). Proteins were then extracted using an extraction buffer (4% SDS, 100 mM Tris-HCl, pH 7.6). Protein concentration was determined using the BCA Protein Assay Kit (Bio-Rad, USA). To evaluate protein integrity and ensure equal loading across samples, a quality control (QC) step was performed: 20 µg of protein from each sample was mixed with 5× loading buffer, boiled at 95 °C for 5 min, and resolved by SDS-PAGE (4%-20% precast gradient gel) at 180 V for 45 min. The gel was stained with Coomassie Brilliant Blue R-250 to visualize the protein profile (**Supplementary Figure 1**). The electrophoretic profiles revealed clear and well-resolved protein bands without significant degradation. The high degree of consistency in band distribution and intensity across all samples confirmed accurate protein quantification and uniform loading, ensuring that the concentration and total protein amount were sufficient for downstream mass spectrometry analysis.

Protein digestion was performed according to the Standard Operating Procedure (SOP) for urinary proteomics (Wiśniewski et al., 2009) using the Filter-aided Sample Preparation (FASP) method (Liu et al., 2025). Briefly, 20 µg of protein per sample was reduced with 100 mM DTT at 95 °C for 5 min and subsequently alkylated with 20 mM iodoacetamide (IAA) in the dark for 30 min. The protein mixture was then transferred into a 10 kDa MWCO filter unit (Microcon, Millipore) and washed three times with UA buffer (8 M urea, 0.1 M Tris-HCl, pH 8.5) to remove SDS and other small molecule contaminants. Digestion was carried out using trypsin (Promega) at a protein-to-enzyme ratio of 1:50 (wt/wt) at 37 °C for 16 h.A “Pool” sample was generated by combining equal protein amounts from all individual samples for spectral library construction. Peptides from the Pool sample were fractionated into 10 fractions using the High pH Reversed-Phase Peptide Fractionation Kit (Pierce, Thermo Scientific). All peptides were desalted using C18 cartridges (Empore), lyophilized, and reconstituted in 0.1% formic acid. Peptide concentrations were measured by UV absorbance at 280 nm. Indexed retention time (iRT) calibration peptides (Biognosys) were added to both Pool and individual sample peptides prior to mass spectrometry analysis.

#### 2.4.2 Mass spectrometry assay

All fractionated and individual samples were analyzed using a timsTOF Pro mass spectrometer (Bruker, USA) interfaced with an Evosep One system (Evosep, Denmark).For spectral library generation, the mass spectrometer was operated in Data-Dependent Acquisition (DDA) mode with PASEF (Parallel Accumulation-Serial Fragmentation). The accumulation and ramp time were set to 100 ms each. Mass spectra were acquired in the range of m/z 100–1700 in positive electrospray mode. The ion mobility (1/K0) was scanned from 0.75 to 1.35 Vs/cm², followed by 10 PASEF MS/MS scans per cycle (with a total cycle time of 1.1 s). The dynamic exclusion was set to 24.0 s to prevent repeated sequencing of the same precursor. The ion source voltage was maintained at 1500 V, with a dry gas flow of 3 L/min at 180 °C.For individual sample analysis, the system was operated in Data-Independent Acquisition (DIA) mode. The mass spectrometer collected ion mobility MS spectra over a mass range of m/z 100-1700. Up to 4 windows were defined for each 100 ms TIMS scan based on the m/z-ion mobility plane. During MS/MS scanning, the collision energy was ramped linearly as a function of mobility, ranging from 20 eV at 1/K0 = 0.85 Vs/cm² to 59 eV at 1/K0 = 1.30 Vs/cm².

#### 2.4.3 Mass spectrometry data analysis

DDA raw files were processed using Spectronaut™ (version 14.4.200727.47784, Biognosys, Switzerland) for spectral library construction. The MS/MS spectra were searched against the UniProtKB Homo sapiens database (Taxon ID: 9606, accessed in January 2024), which contained 204,318 sequences. To calibrate retention time, the iRT peptide sequences (iRT Kit, Biognosys) were incorporated into the FASTA database. The search parameters were configured as follows: enzyme, trypsin; maximum missed cleavages, 2; fixed modification, carbamidomethyl (C); and dynamic modifications, oxidation (M) and protein N-term acetylation. Protein identification was filtered using a false discovery rate (FDR) threshold of ≤ 1%.DIA data were analyzed using Spectronaut™ by searching against the previously constructed spectral library. The analysis parameters included: retention time prediction, dynamic iRT; interference correction at the MS2 level, enabled; and cross-run normalization, enabled. All results were filtered based on a Q-value cutoff of 0.01 (equivalent to FDR < 1%). The final list of identified proteins and peptides is provided in **Appendix 1**.

### 2.5 Detection of urinary TM and plasma TM

“Peripheral venous blood was collected from sepsis patients and healthy controls within 2 hours of admission into sodium citrate anticoagulant tubes (1:9 ratio), while midstream urine was collected in standard sterile tubes. All specimens were processed within 1 hour of collection. Plasma was isolated by centrifugation at 3000 rpm for 10 minutes at room temperature. TM concentrations in both plasma and urine were determined via chemiluminescent enzyme immunoassay (CLEIA) using the HISCL Thrombomodulin Assay Kit (Sysmex Corporation, Kobe, Japan; NMPA Registration No. 20152403877) on a HISCL-800 automated analyzer. The procedures were performed strictly in accordance with the manufacturer’s standardized protocol for the TM assay, including automated luminescence quantification and system calibration.”

### 2.6 Urinary TM colloidal gold test

#### 2.6.1 Synthesis of the colloidal gold-labeled mAb

Colloidal gold was prepared via the sodium citrate reduction method. Initially, 1 mL of 0.1% HAuCl4 solution was mixed with 99 mL of ultrapure water and brought to a boil with continuous stirring. Upon boiling, 2 mL of 1% sodium citrate was introduced, turning the solution wine-red, and the mixture was heated for an additional 6 minutes. The resulting colloidal gold solution was cooled and stored at 4 °C. For conjugation, 5 mL of colloidal gold solution was transferred to a 15 mL centrifuge tube, and the pH was adjusted to 8.2 using 0.2 M K2CO3. The anti-TM monoclonal antibody was diluted to 0.2 mg/mL in 0.02 M borate buffer. Subsequently, 10 μL of this diluted antibody was added to the 5 mL colloidal gold solution and incubated for 45 minutes. To block any unbound sites, 0.25 mL of a 10% BSA solution was added, and the mixture was further incubated for 2 hours. The conjugate was then centrifuged at 8000 rpm at 4 °C for 45 minutes. The supernatant was removed, and the precipitate was resuspended in 0.5 mL of gold conjugate suspension. The gold-labeled antibody was stored at 4 °C. This method has currently applied for a Chinese invention patent (No. CN118130788A, **Supplementary File**).

#### 2.6.2 The method of utilizing urinary TM test strip

Dispense 40 μL of the resuspended colloidal gold-labeled solution into a well of a 96-well plate. Subsequently, introduce 10 μL of the colloidal gold-labeled antibody into the same well and ensure thorough mixing by pipetting up and down. Withdraw 10 μL of the supernatant from the urine sample that has been left to stand and add it to the well, followed by pipetting up and down to mix. Incubate the mixture for 5 minutes. Post incubation, add 80 μL of loading buffer and mix well by pipetting. Subsequently, transfer 120 μL of the reacted solution into the sample-application well of the test strip, initiate timing, and after 10 minutes, observe the results. It is imperative to interpret the results within 1 minute of the timing cessation; any interpretations made beyond this time frame are deemed invalid.

### 2.7 Statistical methods

The sample size calculation was performed using PASS 11 software, and clinical data analysis was conducted using SPSS 27.0 statistical software. Categorical variables were expressed as counts (percentages), and intergroup comparisons were conducted using the chi-square (χ²) test. The normality of continuous variables was assessed using the Shapiro-Wilk test. Continuous variables with normal distribution were presented as mean ± standard deviation (SD), whereas those with non-normal distribution were reported as median (interquartile range) [M (Q1, Q3)]. For parametric comparisons, two-group comparisons of normally distributed and homoscedastic data were performed using Student’s t-test. One-way ANOVA followed by Tukey’s *post-hoc* test was employed for multi-group comparisons to determine pairwise differences when a significant overall effect was observed. For non-parametric comparisons, the Mann-Whitney U test was used for two-group comparisons. The Kruskal-Wallis test was applied for multi-group comparisons, with significant results followed by Dunn’s test for pairwise comparisons.

Missing value imputation (k-nearest neighbors (KNN) algorithm), Principal Component Analysis (PCA), hierarchical clustering (Euclidean distance), identification of differentially expressed proteins (DEPs) (|log_2_ fold change (FC)| > 1, p < 0.05), and Gene Ontology (GO) enrichment were performed using Omicsolution (https://wkomics.omicsolution.com/wukong/NAguideR/), while Kyoto Encyclopedia of Genes and Genomes (KEGG) pathway analysis was conducted via CNSknowall (https://cnsknowall.com/#/Home/HighAll). These platforms are integrated R-based web environments tailored for high-throughput proteomic data processing and statistical mining. The STRING website was used for protein-protein interaction (PPI) network analysis and visualization of relevant enrichment. Machine learning analyses were performed using the e1071 and Boruta packages in R version 4.4.2. GraphPad Prism statistical software was used to perform receiver operating characteristic (ROC) curve analysis. The area under the curve (AUC) value was utilized to assess the clinical utility of the biomarker and compare it with C-reactive protein (CRP) and procalcitonin (PCT). Statistical significance was defined as a two-tailed P-value < 0.05.

## 3 Result

### 3.1 Urinary TM as a DEP identified through 4D proteomics

**Figure 2:**
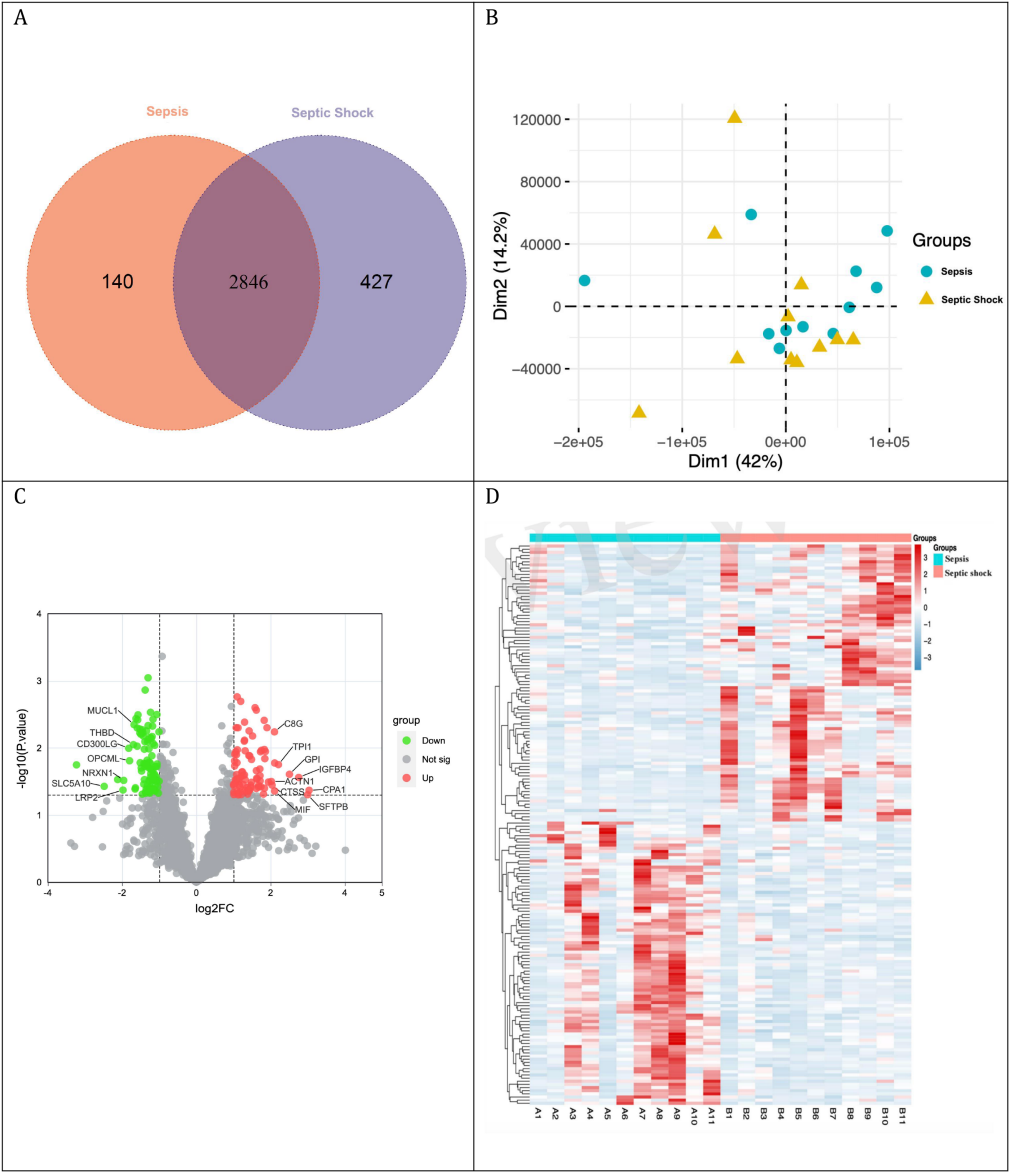
Comparative analysis of protein expression profiles. **(A)** Venn diagram: Overlapping proteins in sepsis and sepsis shock groups; **(B)** Principal component analysis; **(C)** Volcano plot: upregulated proteins in red and downregulated proteins in green; **(D)** DEPs clustering analysis.

In this proteomics study, 22 patients were evenly divided into a sepsis group and a septic shock group, each with 11 participants. Comparative analysis revealed significant differences in critical biomarkers, including WBC, ANC, PT, creatinine, lactate, APACHE II score and SOFA score (**Table 1**). Mass spectrometry analysis of urine samples from 11 patients with sepsis and 11 with septic shock revealed the identification of 4554 distinct proteins. Upon stringent exclusion criteria of proteins with missing values exceeding 50%, a comparative Venn diagram analysis elucidated a core set of 2846 proteins that were common to both sepsis and septic shock cohorts (**Figure 2A**). PCA distinctly segregates the two groups in the reduced-dimensional space, exhibiting compact clustering within each group, which underscores the substantial and consistent differences in their protein expression profiles. (**Figure 2B**). A volcano plot graphically represented 178 DEPs, comprising 88 upregulated and 90 downregulated proteins.DEPs were identified using a threshold of |log_2_ fold change| > 1 and a p-value < 0.05 (**Figure 2C**). Hierarchical clustering analysis of DEPs delineated two distinct clusters, segregating the sepsis and septic shock groups. This finding underscores the substantial divergence in protein expression profiles between these two clinical entities (**Figure 2D**).

**Table 1:** Baseline characteristics of patients in the proteomics study.

Parameters	Sepsis (N=11)	Septic shock (N = 11)	P value
Age, yr	85.0 [58.0, 87.0]	82.0 [72.0, 83.0]	0.186
Male, n (%)	8(72.7%)	4(36.4%)	0.087
WBC,×10^9^/L	9.3 (3.6)	17.4 (4.9)	<0.001
ANC,×10^9^/L	7.7 (3.4)	15.2 (4.3)	<0.001
ALC, ×10^9^/L	0.9 (0.5)	0.9 (0.6)	0.847
RBC, ×10^12^/L	3.4 (0.9)	3.4 (1.1)	0.967
Platelet,×10^9^/L	110.0 [90.0, 171.0]	34.0 [64.0, 92.0]	0.009
PT, s	15.7 (1.6)	18.8 (1.3)	<0.001
APTT, s	35.6 [30.0, 43.9]	35.8 [30.1, 68.3]	0.450
Fibrinogen, g/L	2.9 (0.9)	2.8 (0.9)	0.879
D-Dimer	1.9 [1.4, 3.8]	2.6 [1.3, 4.3]	0.622
ALT, U/L	29.5 [25.3, 48.4]	33.4 [26.7, 56.8]	0.643
AST, U/L	43.9 [29.1, 63.2]	45.7 [30.7, 70.3]	0.533
TBIL, mmol/L	19.0 [10.2, 24.5]	23.2 [11.3, 25.8]	0.775
Albumin, g/L	29.8 (3.2)	27.0 (3.7)	0.074
Creatinine, mol/L	76.6 (21.9)	168.7 (44.2)	<0.001
CRP, g/L	67.9 (46.4)	132.6 (100.2)	0.042
Lactate, mmol/L	1.4 [1.1, 1.5]	3.1 [2.6, 4.4]	<0.001
APACHE II score	18.6 (2.4)	25.8 (2.7)	<0.001
SOFA score	6.0 [6.0, 7.0]	11.0 [10.0, 13.0]	<0.001

**Figure 3:**
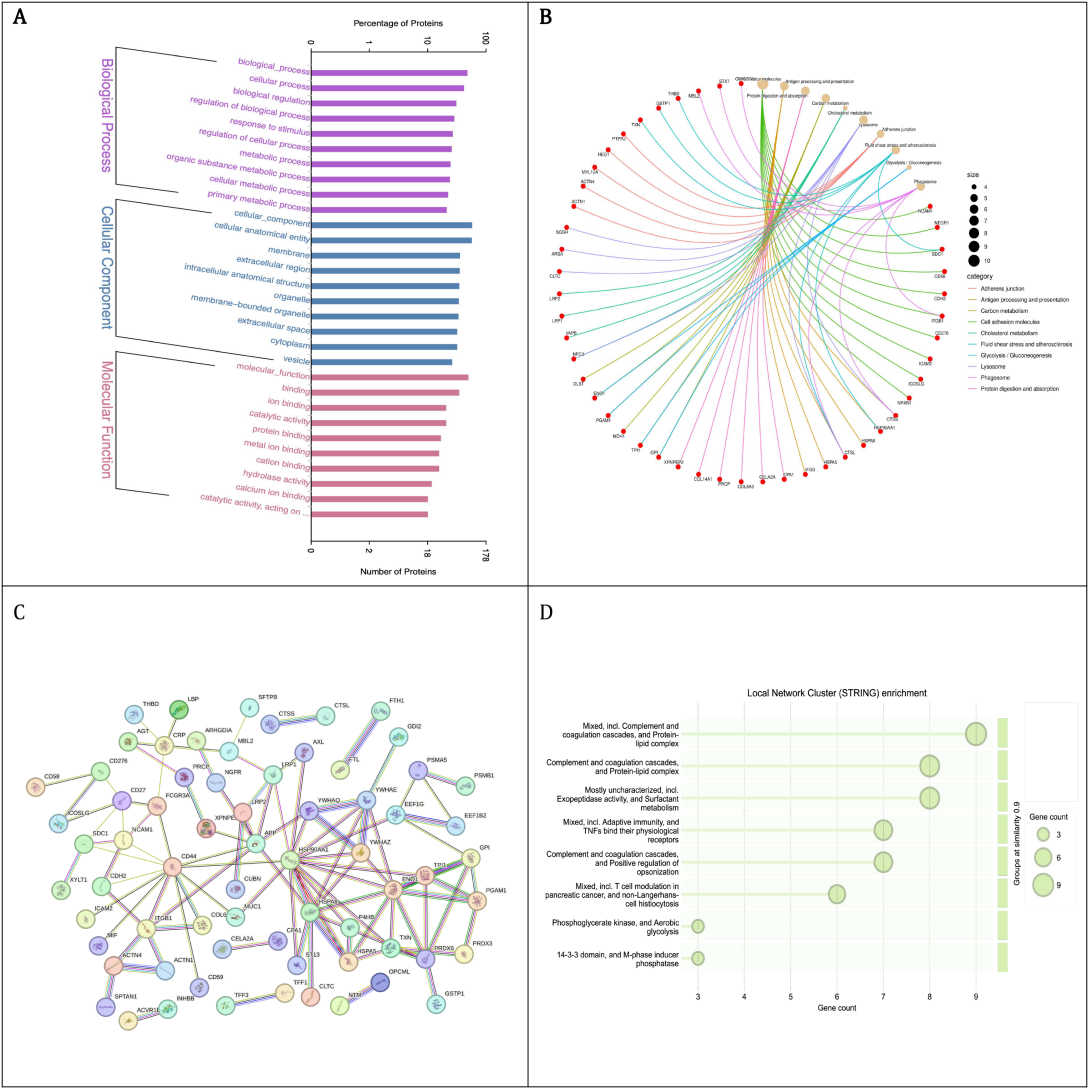
Biological function analysis of DEPs. **(A)** GO functional annotation; **(B)** KEGG enrichment; **(C)** PPI network analysis with a stringency threshold of 0.7; **(D)** Functional enrichment of local network clusters.

GO functional annotation of 178 DEPs revealed predominant involvement in biological processes, notably in response to cellular stimuli and the regulation of cellular processes. In terms of cellular components, these DEPs were predominantly localized to the extracellular region. Concerning molecular functions, the DEPs were primarily associated with biomolecule interactions and catalytic activities (**Figure 3A**). KEGG enrichment analysis indicated significant enrichment of DEPs in pathways related to cell adhesion molecules, lysosomes, and metabolism-related pathways (**Figure 3B**). PPI network analysis was performed on the differentially expressed proteins, followed by enrichment analysis of the network. The results highlighted that the PPI network was predominantly enriched in pathways associated with immune response and coagulation (**Figures 3C, D**).

**Figure 4:**
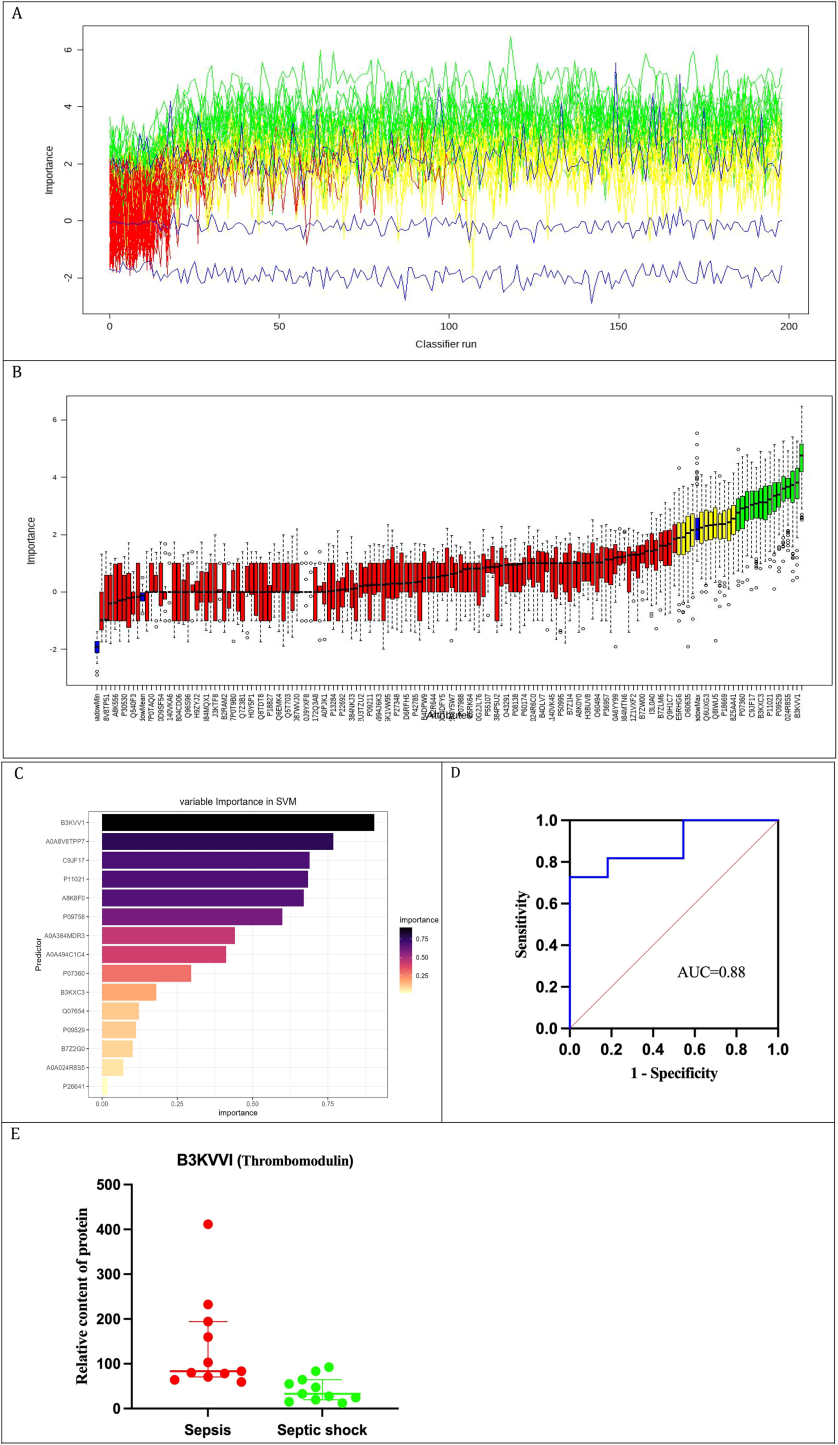
Screening of biomarkers from DEPs using machine learning methods. **(A)** Dynamic change plot of feature importance based on 200 Boruta algorithm iterations; **(B)** Feature importance ranking plot (green denotes important variables, while red/blue/yellow indicate rejected variables); **(C)** Variable importance ranking in SVM; **(D)** ROC curve of TM for diagnosing septic shock. **(E)** The relative content of TM protein (Median with interquartile range).

To identify clinically relevant diagnostic biomarkers, a preliminary screen of 178 DEPs was performed. Following the exclusion of immunoglobulins lacking gene names, the Boruta algorithm, leveraging Random Forest, was employed for feature selection (**Figures 4A, B**). This process culminated in the selection of 15 key variables by Boruta, which were then analyzed using SVM analysis. Among these, B3KVV1 (Thrombomodulin) emerged as the most significant (**Figure 4C**). The diagnostic accuracy for septic shock, as indicated by the area under the ROC curve, was 0.88 (**Figure 4D**). The concentration of TM in the urine of sepsis patients is notably elevated compared to that observed in patients with septic shock (**Figure 4E**).

### 3.2 Diagnostic value of urinary TM in patients with sepsis and septic shock

To ascertain the diagnostic utility of urinary thrombomodulin (TM) in sepsis, we recruited 83 individuals with sepsis and 31 with septic shock, using 50 healthy volunteers as a control group. As depicted in **Table 2**, in contrast to the healthy controls, sepsis patients demonstrated elevated white blood cell counts, ALT, AST, fibrinogen, and D-dimer levels, alongside reduced red blood cell count, platelet count, albumin levels, and antithrombin activity, with extended PT and APTT (P<0.001). Furthermore, when compared with sepsis patients, those experiencing septic shock presented with higher white blood cell counts, creatinine, lactate levels, SOFA and APACHE II scores, along with a greater incidence of AKI and a higher 28-day mortality rate (P<0.01).

**Table 2:** Baseline characteristics for urinary TM in sepsis and septic shock patients compared to healthy controls.

Parameters	Healthy controls (N=50)	Sepsis (N = 83)	Septic shock (N = 31)	P value
Age,yr	71.0 [66.0, 76.0]	72.0 [60.0, 83.0]	82.0 [61.0, 86.0]	NS^a,b,c^
Male,n(%)	29 (58.0%)	54 (65.1%)	19 (61.3%)	NS*
SOFA score	-	5 [4, 7]	9 [7, 12]	0.001^c^
APACHE II score	-	20 [15, 25]	27 [21, 30]	0.001^c^
Inflammation markers				
Procalcitonin,g/ml	-	0.4 [0.2, 1.8]	1.4 [0.6, 5.3]	0.010^c^
CRP, g/L	-	54.8 [24.4, 103.3]	89.3 [56.2, 130.4]	0.032^c^
Complete blood count				
WBC,×10^9^/L	6.0 [5.0, 7.1]	8.4 [6.1, 13.0]	11.7 [9.9, 15.1]	0.001^a,b^ 0.002^c^
ANC,×10^9^/L	3.5 [2.7, 4.3]	6.6 [4.8, 11.3]	10.0 [8.2, 14.0]	0.001^a,b^ 0.005^c^
ALC, ×10^9^/L	1.9 [1.6, 2.1]	0.8 [0.6, 1.2]	0.7 [0.4, 1.1]	0.001^a,b^ NS^c^
RBC, ×10^12^/L	4.7 (0.6)	3.4 (0.9)	3.5 (1.1)	0.001^a,b^ NS^c^
Hemoglobin, g/L	134.0 (20.7)	100.0 (23.0)	102.0(31.9)	0.001^a,b^ NS^c^
Platelet,×10^9^/L	232.5 (67.7)	166.00 (80.5)	151.00 (75.4)	0.001^a,b^ NS^c^
Organ function				
ALT, U/L	15.0 [12.0, 25.75]	30.1 [16.9, 55.8]	36.8 [16.3, 56.2]	0.001^a^ 0.005^b^ NS^c^
AST, U/L	19.0 [16.0, 23.0]	39.5 [24.9, 66.2]	41.4 [26.6, 74.3]	0.001^a,b^ NS^c^
TBIL, mmol/L	12.3 [8.9, 16.0]	12.6 [8.8, 16.7]	12.9 [9.2, 17.8]	NS^a,b,c^
Total protein, g/L	66.3 [63.6, 71.7]	56.9 [52.9, 62.1]	56.8 [50.9, 63.7]	0.001^a,b^ NS^c^
Albumin, g/L	42.9 [41.6, 46.3]	31.8 [29.0, 36.3]	30.8 [28.9, 33.6]	0.001^a,b^ NS^c^
Creatinine,μmol/L	67.0 [60.8, 84.0]	71.6 [66.5, 108.0]	100.2 [79.1, 166.8]	NS^a^ 0.002^b,c^
NT-proBNP, pg/ml	-	991.3 [391.2, 3634.4]	2389.6 [572.6, 8647.4]	0.024^c^
Lactate, mmol/L	-	1.50 [1.0, 1.8]	2.60 [2.3, 5.2]	0.001^c^
Coagulation test				
PT, s	11.4 [11.0, 11.7]	14.0 [12.6, 15.3]	14.0 [12.9, 16.6]	0.001^a,b^ NS^c^
INR	0.97 [0.95, 1.00]	1.16 [1.05, 1.27]	1.18 [1.08, 1.38]	0.001^a,b^ NS^c^
APTT, s	27.7 [26.3, 29.0]	32.0 [27.8, 39.7]	30.0 [28.0, 37.2]	0.001^a^ 0.005^b^ NS^c^
Fibrinogen, g/L	2.74 [2.3;3.3]	3.9 [3.0, 4.5]	3.0 [2.0, 4.2]	0.001^a^ NS^b^ 0.004^c^
TT, s	16.7 [15.9, 17.0]	15.6 [14.5, 16.8]	16.30 [15.7, 18.6]	0.001^a^ NS^b^ 0.044^c^
D-dimer, mg/L	0.2 [0.2, 0.3]	1.82 [0.9, 3.5]	2.77 [1.2, 6.6]	0.001^a,b^ NS^c^
Antithrombin, %	86.9 [78.7, 95.7]	73.0 [55.6, 88.0]	66.1 [44.0, 91.0]	0.001^a,b^ NS^c^
Prognosis				
Incidence of AKI (%)	-	27.7 (23)	51.6 (16)	0.017*
28-day ICU mortality rate (%)	-	10.8 (9)	54.8 (17)	0.001*

^a^Comparison between the healthy control group and the general sepsis group; ^b^Comparison between the healthy group and the septic shock group; ^c^Comparison between the sepsis group and the septic shock group; *Chi-square test.

**Figure 5:**
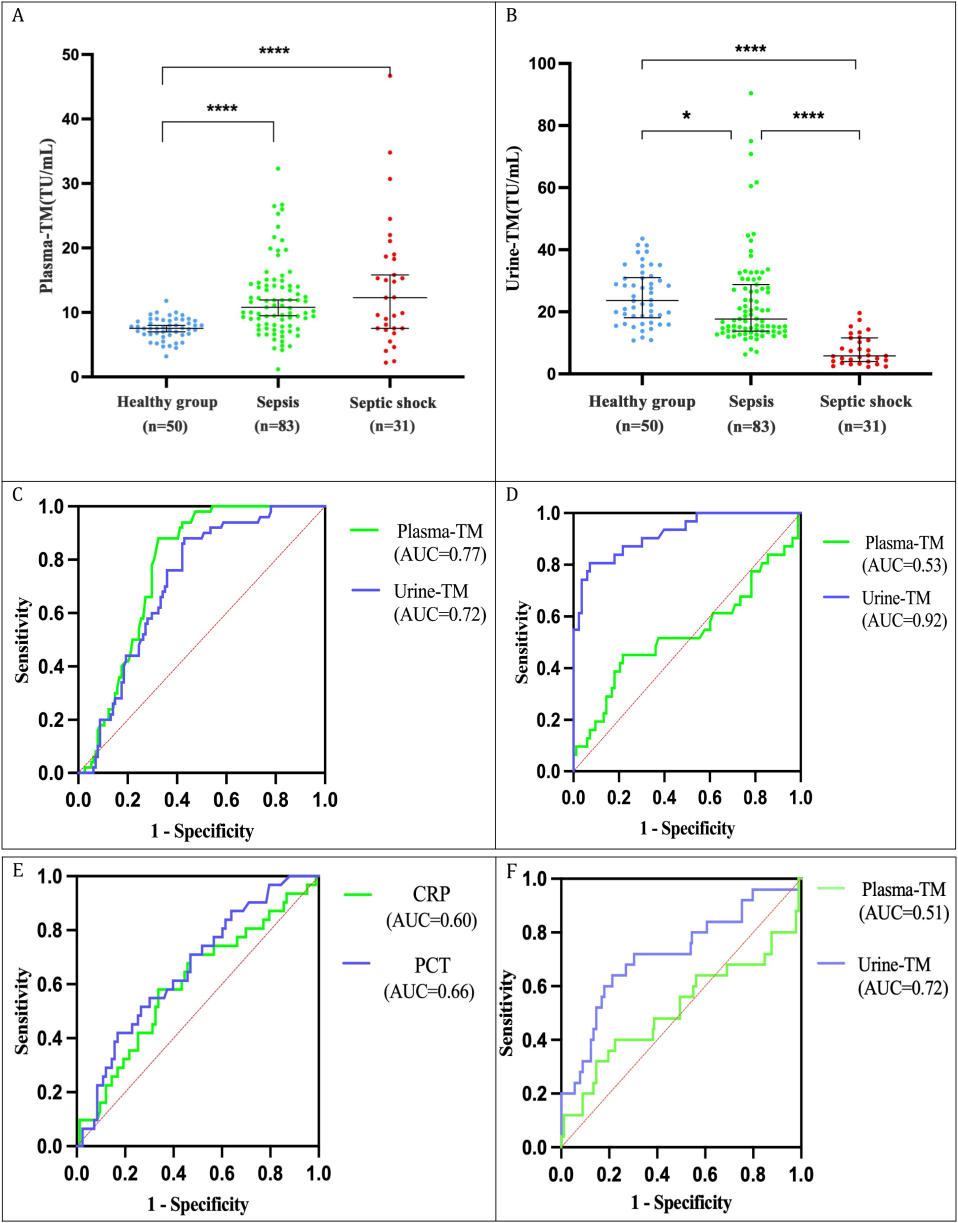
Comparative analysis of plasma TM and urine TM levels and their diagnostic efficacy in sepsis and septic shock. **(A)** Expression levels of plasma TM; **(B)** Expression levels of urinary TM; **(C)** ROC curves for plasma TM and urinary TM in sepsis diagnosis (P = 0.30); **(D)** ROC curves for plasma TM and urinary TM in septic shock diagnosis (P<0.001); **(E)** ROC curves for CRP and procalcitonin (PCT) in septic shock diagnosis; **(F)** ROC curves for plasma TM and urinary TM in predicting 28-Day mortality in sepsis (P = 0.029). *P < 0.05; ****P < 0.0001.

Plasma and urinary TM concentrations were measured using chemiluminescent immunoassay. Plasma TM levels significantly increased across the spectrum of disease severity, from the healthy control group to the sepsis and septic shock groups (p<0.05) (**Figure 5A**). Conversely, urinary TM levels significantly decreased (p<0.05) (**Figure 5B**). Compared with sepsis, septic shock patients exhibited a significant decrease in urinary TM levels (p<0.05), while the increase in plasma TM levels was not significant (p>0.05). The ROC curve analysis indicated that the diagnostic performance of urinary TM for sepsis at a threshold of 15.46 TU/ml [area under the ROC curve (AUC)=0.72, sensitivity 57.0%, specificity 88.0%] was comparable to that of plasma TM at a threshold of 9.04 TU/ml (AUC = 0.77, sensitivity 67.5%, specificity 88.0%), with no statistically significant difference (P = 0.30) (**Figure 5C**). However, for septic shock, urinary TM at a threshold of 11.85 TU/ml demonstrated superior diagnostic performance (AUC = 0.92, sensitivity 92.8%, specificity 80.6%) compared to plasma TM (AUC = 0.53, sensitivity 45.2%, specificity 78.3%), CRP (AUC = 0.60), and PCT (AUC = 0.66), with a statistically significant difference (P<0.001) (**Figures 5D, E**). Furthermore, urinary TM at 11.85 TU/ml was significantly more effective in predicting 28-day mortality in sepsis patients (AUC = 0.72, sensitivity 60.0%, specificity 82.0%) than plasma TM (AUC = 0.51, sensitivity 77.5%, specificity 40.0%) (P = 0.029) (**Figure 5F**).

**Figure 6:**
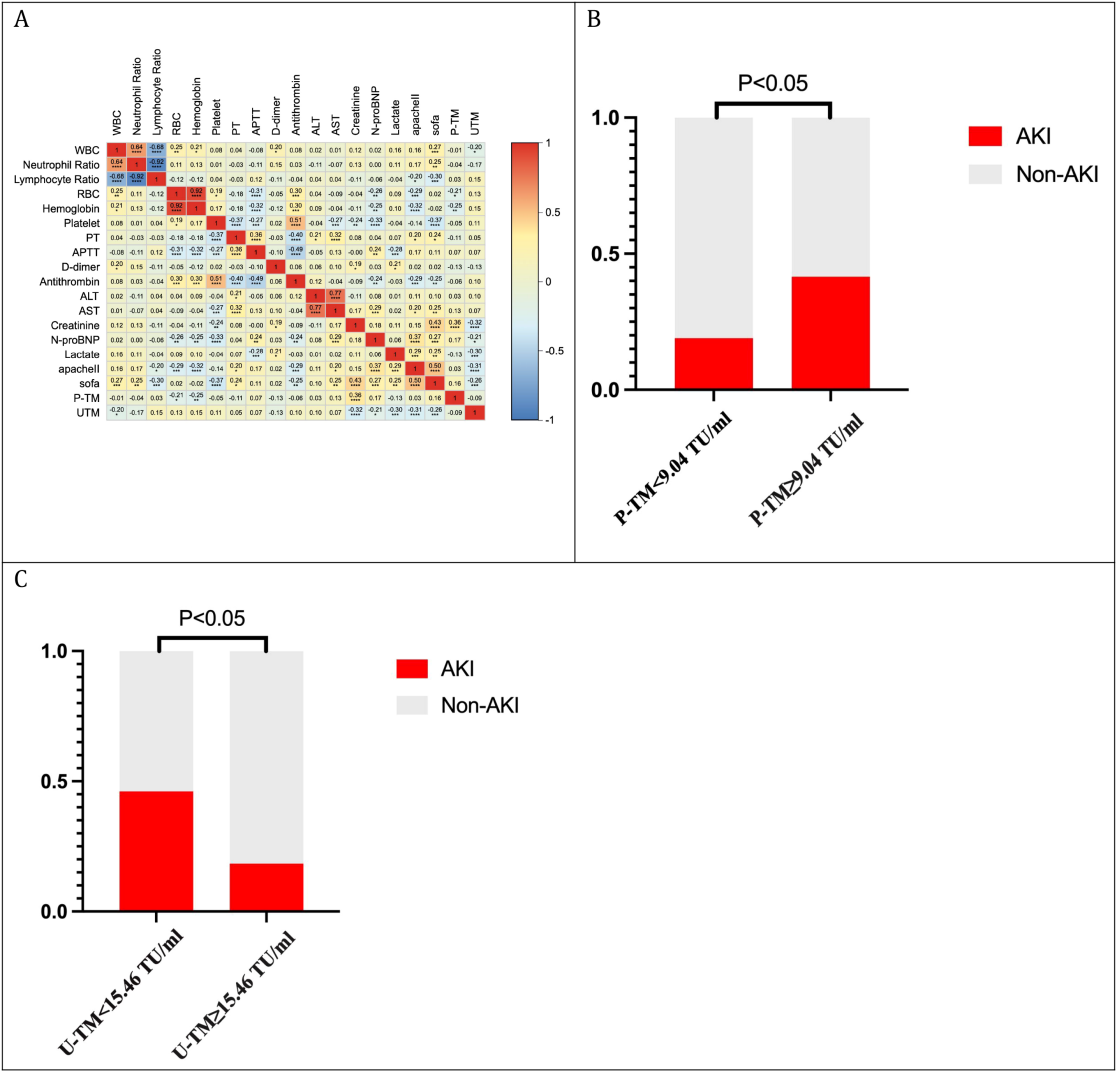
Correlation analysis of TM and subgroup analysis in sepsis. **(A)** Correlation analysis; **(B)** Relationship between plasma TM and incidence of sepsis-associated AKI; **(C)** Relationship between urinary TM and incidence of sepsis-associated AKI.

Correlation analysis revealed that plasma TM levels were positively correlated with creatinine (r=0.36), negatively correlated with RBC (r=-0.21), and negatively correlated with hemoglobin (r=-0.25). Urinary thrombomodulin (U-TM) levels were negatively correlated with creatinine (r=-0.32), NT-proBNP (r=0.21), lactate (r=-0.30), SOFA score (r=-0.31), and APACHE II score (r=-0.26) (all P<0.05) (**Figure 6A**). Due to the significant association between plasma and urinary TM levels and creatinine concentration, a comparative analysis of the incidence of sepsis-induced AKI was conducted. The results indicated that the incidence of AKI in sepsis patients with plasma TM levels of 9.04 TU/mL or higher was significantly higher than in those with P-TM levels below 9.04 TU/mL (41.6% vs. 18.9%) (**Figure 6B**); similarly, the incidence of AKI in sepsis patients with urinary TM levels below 15.46 TU/mL was significantly higher than in those with urinary TM levels of 15.46 TU/mL or higher (46.2% vs. 18.4%) (**Figure 6C**).

### 3.3 Development and validation of a colloidal gold test strip for urinary TM

**Figure 7:**
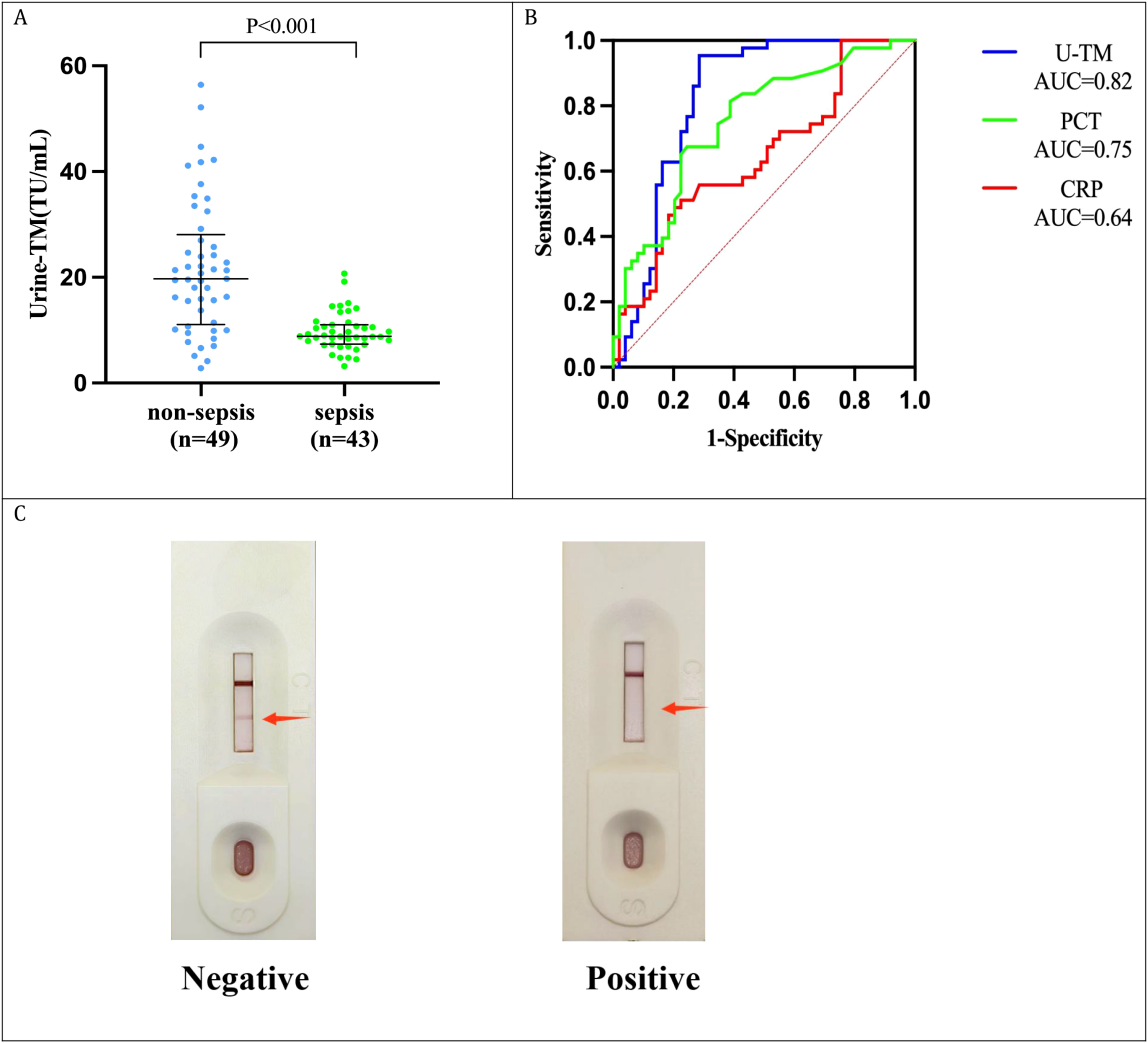
Validation of a colloidal gold test strip for urinary TM. **(A)** Comparative Urinary TM Expression in sepsis (n=43) and non-sepsis (n=49) ICU Patients; **(B)** ROC Analysis of urinary TM, plasma CRP, and plasma PCT for diagnosing sepsis; **(C)** Our developed urine TM colloidal gold test card: No color change post-urine addition signifies a positive result (urine TM <15.46 TU/ml, indicating sepsis); color change indicates a negative result (urine TM >15.46 TU/ml, no sepsis). The arrow indicates whether there is a change in the band color.

We enrolled 92 ICU patients who were receiving treatment, with 43 of them diagnosed with sepsis based on the Sepsis-3.0 criteria. The demographic and clinical characteristics of the two cohorts are detailed in **Table 3**. Chemiluminescence immunoassay analysis of urine samples from these patients revealed that patients without sepsis had urinary TM levels of 19.69 (11.07-28.09) TU/mL, whereas those with sepsis had significantly lower levels of 8.83 (7.34-11.00) TU/mL (**Figure 7A**). The AUC for diagnosing sepsis using urinary TM was 0.82, with an optimal diagnostic cutoff of 15.29 TU/mL, yielding a sensitivity of 95.3% and a specificity of 71.4%, which surpassed the diagnostic accuracy of concurrently measured plasma CRP and PCT (**Figure 7B**).

**Table 3:** Baseline characteristics of patients in the colloidal gold test.

Parameters	Non-sepsis (*N=49)*	Sepsis *(N = 43)*	P value
Age, yr	78.0 [58.0, 86.0]	84.0 [68.0, 88.0]	0.181
Male, n(%)	35(71.4%)	28(52.8%)	0.423
Procalcitonin, g/ml	0.1 [0.1, 0.3]	0.5 [0.2, 2.6]	<0.001
CRP, g/L	29.2 [12.7, 54.2]	58.6 [17.1, 106.7]	0.023
WBC,×10^9^/L	8.7 [7.6, 12.4]	11.9 [9.0, 14.3]	0.008
ANC,×10^9^/L	6.9 [5.4, 10.7]	9.1 [7.2, 11.5]	0.011
ALC, ×10^9^/L	1.3 [0.9, 1.7]	1.2 [0.7, 1.5]	0.562
RBC, ×10^12^/L	3.6 (0.9)	3.0 (0.8)	0.001
Hemoglobin, g/L	105.0 [83.0, 131.0]	91.0 [71.0, 98.5]	<0.001
Platelet,×10^9^/L	247.2 (80.9)	211.3 (98.9)	0.062
ALT, U/L	23.0 [12.6, 39.8]	24.0 [12.7, 43.6]	0.628
AST, U/L	30.0 [20.6, 45.0]	33.0 [27.3, 47.9]	0.166
Creatinine, mol/L	90.0 [71.2, 102.0]	156.30 [96.4, 221.0]	<0.001
PT, s	13.4 [12.6, 13.8]	13.4 [13.0, 14.6]	0.065
APTT, s	24.6 [22.9, 26.2]	29.3 [25.1, 35.5]	<0.001
Fibrinogen, g/L	5.2 [3.6, 6.1]	4.6 [2.9, 5.8]	0.198

Leveraging the non-invasive, precise, cost-effective, and blood conservation advantages of urinary TM in sepsis diagnosis and prognostic assessment, we partnered with the Suzhou Institute of Biomedical Engineering and Technology at the Chinese Academy of Sciences to develop a colloidal gold-based urine TM test strip. The test strip turns red when the concentration of urinary TM exceeds 15.46 TU/mL, indicating a high level of urinary TM and a reduced probability of sepsis; the absence of color on the strip suggests urinary TM levels below 15.46 TU/mL, which may indicate a sepsis event (**Figure 7C**). This urine TM colloidal gold test strip is suitable for rapid sepsis screening in clinical practice. Applying this test strip to the 92 ICU patients, we identified 48 cases of sepsis, achieving a sensitivity of 86.1%, specificity of 77.6%, and overall accuracy of 81.5%. Statistical analysis using the Kappa test and McNemar’s test revealed no significant difference in diagnostic performance for sepsis between the urine TM test strip and chemiluminescence immunoassay for urinary TM (P>0.05) (**Table 4**).

**Table 4:** Comparison of diagnostic efficacy between chemiluminescent immunoassay and colloidal gold test.

Method	Sepsis-3.0		Sensitivity (95% CI)	Specificity (95% CI)	PPV (95% CI)	NPV (95% CI)	Accuracy (95% CI)	Kappa Test	McNemar’s test	
	Positive	Negative							χ²	p
Chemiluminescent immunoassay										
positive	41	14	95.3% (82.9%~99.2%)	71.4% (56.5%~83.0%)	74.6% (60.7%~84.9%)	94.6% (80.5%~99.1%)	82.6% (73.3%~89.7%)	0.65	–	–
negative	2	35								
Colloidal gold test										
positive	37	11	86.1% (71.4%~94.2%)	77.6% (63.0%~87.8%)	77.1% (62.3%~84.5%)	86.4% (72.0%~94.3%)	81.5% (72.1%~88.9%)	0.63	1.45	0.228
negative	6	38								

The diagnosis of sepsis is based on the Sepsis 3.0 criteria. The chemiluminescent immunoassay identifies a urine TM level of less than 15.46 TU/mL as a positive result, suggesting the presence of sepsis; similarly, the colloidal gold test strip interprets the absence of color development as a positive result, also indicative of sepsis. Kappa test results demonstrate a high degree of concordance between the diagnostic outcomes of the chemiluminescent immunoassay and the urine colloidal gold test strip methods with the Sepsis 3.0 criteria. The McNemar test reveals no statistically significant difference in diagnostic accuracy for sepsis between the chemiluminescent immunoassay and the urine colloidal gold test strip methods.

## 4 Discussion

This study introduces a clinically applicable method for the rapid and accurate screening of sepsis using urine test strips, thereby enhancing diagnostic efficiency and minimizing iatrogenic blood loss in patients. In our research, we utilized 4D-DIA proteomics to examine urine samples from 22 individuals with sepsis, identifying urinary TM. Clinical studies involving 114 sepsis patients and 50 controls confirmed that urinary TM surpasses plasma TM in septic shock diagnosis (P<0.001) and matches its efficacy in sepsis detection (P = 0.30). We also compared urine from 43 sepsis and 49 non-sepsis patients using colloidal gold and Chemiluminescent Immunoassay, finding no significant accuracy difference between the urinary TM strip and immunofluorescence (P = 0.23), suggesting that the urinary TM strip is a dependable non-invasive tool for sepsis screening.

In this proteomics investigation, we identified a total of 4,554 proteins, ensuring the detection of low-abundance proteins and validating the comprehensiveness and reliability of our findings (Zhao et al., 2017). GO functional annotation and KEGG pathway enrichment analysis revealed 178 DEPs predominantly functioning extracellularly, with roles in molecular interactions, cell adhesion, lysosomes, and metabolic pathways. Shen et al (Shen et al., 2024), in a comparative proteomics study of blood samples from 22 sepsis patients and 10 healthy individuals, identified 174 DEPs primarily associated with inflammation, extracellular matrix metabolism, cell secretion, activation, and immune responses. The strikingly similar pathway enrichment profiles between our study and theirs confirm that urinary protein profiles mirror the pathophysiological changes during sepsis. PPI network functional enrichment analysis highlighted the significance of complement and coagulation pathways, aligning with the pathways implicated in the 87 DEPs identified by Palmowski (Palmowski et al., 2025) when distinguishing between sepsis survivors and non-survivors. This suggests that septic shock patients exhibit more severe coagulation and immune dysregulation compared to those with general sepsis, contributing to higher mortality rates (Esmon, 2005; Shankar-Hari et al., 2017). Additionally, we employed a more stringent fold change criterion for DEPs screening than the conventional threshold (Vaes et al., 2014), leveraged the Boruta algorithm to mitigate multicollinearity in proteomics data (Kursa and Witold, 2010), and applied SVM for its robust classification and feature selection capabilities in biomarker identification (Mann et al., 2021). Our results indicate that urinary TM, indicative of endothelial damage, offers superior diagnostic performance for sepsis, with an AUC value of 0.88.

Thrombomodulin, a transmembrane glycoprotein with an approximate molecular weight of 75 kilodaltons, is composed of 559 amino acids and includes an N-terminal epidermal growth factor (EGF)-like domain (Esmon and Owen, 1981). In 1981, Esmon and Owen first isolated TM from rabbit lungs, confirming its capacity to activate protein C efficiently through the formation of a TM-thrombin complex (Suzuki et al., 1987). This activation cascade leads to the inactivation of coagulation factors Va and VIIIa, thereby curbing thrombin generation and conferring anticoagulant properties (Suzuki et al., 1987). TM also plays a role in anti-inflammatory responses by activating protein C (Conway, 2012). TM is found in two distinct forms (Martin et al., 2013): the membrane-bound variant on vascular endothelial cells and the soluble form in body fluids, which arises from the proteolytic cleavage of the membrane-bound TM and is detectable in both blood and urine (Ishii and Majerus, 1985). Kong et al. observed that the release of soluble TM (sTM) from human umbilical vein endothelial cells increased with hydrogen peroxide-induced damage, correlating positively with the duration of exposure to the oxidant (Kong et al., 2017). This implies that elevated sTM levels are indicative of more severe endothelial cell injury. In sepsis, endothelial damage results in the release of membrane-bound TM into the bloodstream, converting it to sTM, which, despite retaining some ability to bind thrombin and activate protein C, is markedly less potent than the membrane-bound form. Consequently, increased sTM levels in the blood of sepsis patients signify vascular endothelial barrier disruption and are linked to a poor prognosis (Watanabe-Kusunoki et al., 2020; Zhou et al., 2022). This study elucidates that, contrary to the escalating plasma TM levels observed with advancing sepsis, urinary TM is more abundant in healthy individuals and diminishes as sepsis severity escalates. Existing literature has documented a positive correlation between plasma TM levels and creatinine levels, in contrast to the negative correlation exhibited by urinary TM levels with creatinine, highlighting the renal metabolism’s influence on TM balance (Aso et al., 1998; Bouchard et al., 2015). Our study further reveals that urinary TM concentration is robustly and inversely correlated with brain natriuretic peptide, lactate levels, APACHE II scores, and SOFA scores, a relationship not observed with plasma TM. This suggests that U-TM levels are a more precise indicator of disease severity than plasma TM levels. Prior research has identified plasma TM as a biomarker for acute kidney injury in sepsis patients (Katayama et al., 2017). Our subgroup analysis of septic renal injury indicates that urinary TM is more effective in identifying sepsis complicated by AKI than plasma TM. Additionally, urinary TM surpasses plasma TM in predicting the 28-day mortality risk in sepsis patients, a novel finding not previously reported in analogous studies.

In our study, we have engineered and fabricated a colloidal gold-based lateral flow assay strip calibrated to a color threshold of 15.46 TU/mL for TM, which exhibits comparable diagnostic accuracy to the immunofluorescence technique in detecting urinary TM. This assay strip leverages a conventional citrate mono-reducing agent system for its preparation. A distinct advantage of this system is the elimination of the necessity for stringent control over the ratios of various reagents, with the reaction culminating in a visually discernible burgundy hue, facilitating quality assurance during large-scale manufacturing processes (Lin et al., 2024). The reagents employed, including citrate, bovine serum albumin (BSA), and borate buffer, are all standard biochemicals, which are more cost-effective compared to precious metal catalysts. The BSA blocking agent demonstrates broad applicability and superior compatibility with the majority of antibodies, surpassing casein and gelatin in this regard, thereby mitigating process variability attributed to antibody batch discrepancies (Daniel and Astruc, 2004). This colloidal gold assay strip negates the need for urine sample pretreatment, simplifying the procedure. It rapidly alters color within a 10-minute interval post-sample introduction, with the color intensity being directly proportional to the urinary TM concentration. The assay outcomes are highly congruent with those ascertained via Chemiluminescent Immunoassay, exhibiting robust resistance to interference. Moreover, the test line (T line) delineates a crisp demarcation between positive and negative results, devoid of any trailing or nonspecific bands, rendering it readily discernible to the unaided eye.

This study acknowledges several limitations. Firstly, it is a single-center observational study with a limited sample size; future research should aim to increase the sample size and consider multi-center prospective cohort studies for enhanced generalizability. Secondly, continuous dynamic monitoring of urinary TM in sepsis patients was not performed, which could provide valuable insights into the temporal dynamics of this biomarker. Thirdly, the sample size for proteomics data was inadequate, potentially limiting the robustness of findings in this domain.

In conclusion, the findings of this study suggest that the urinary TM colloidal gold test strip, characterized by its non-invasive nature, rapid response, and high sensitivity, holds promise as a potent tool for the non-invasive screening of sepsis and for the evaluation of adverse outcomes within a 28-day period.

## Data Availability

The datasets presented in this study can be found in online repositories. The names of the repository/repositories and accession number(s) can be found in the article/**Supplementary Material**.

## Supplementary material

Supplementary Figure 1 The quality of the extracted proteins was assessed using SDS-PAGE.
