# Supplementary material for: From proteomics to colloidal gold tests for urinary thrombomodulin: a prospective cohort study on accurate sepsis screening
Source: Front Cell Infect Microbiol. 2026 Mar 9;16:1779950. doi: 10.3389/fcimb.2026.1779950 (PMC13006627; doi:10.3389/fcimb.2026.1779950)
Supplement: Supplementary Figure 1 — The quality of the extracted proteins was assessed using SDS-PAGE. [file Image1.pdf]

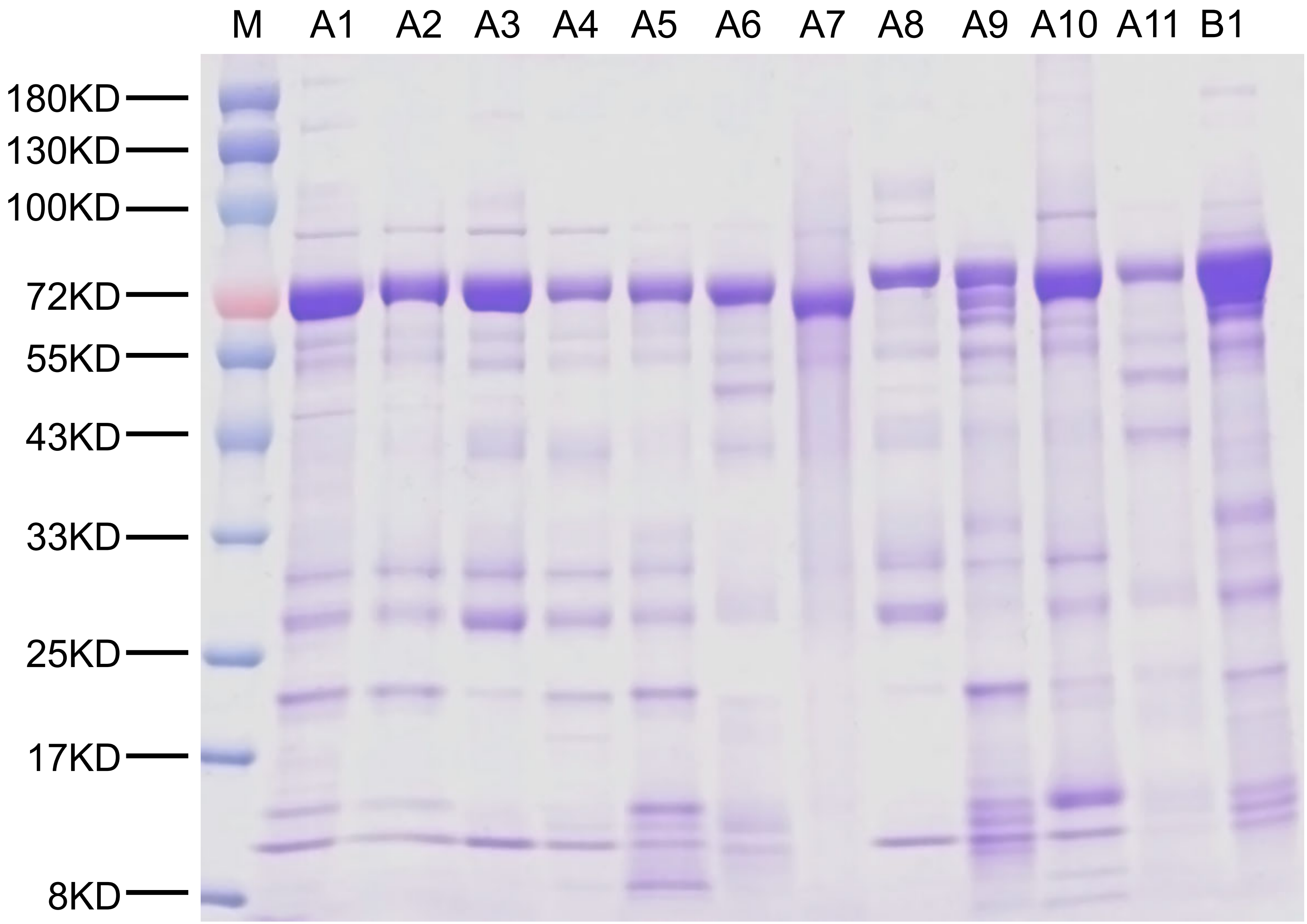
M
A1
A2
A3
A4
A5
A6
A7
A8
A9
A10
A11
B1
180KD
130KD
100KD
72KD
55KD
43KD
33KD
25KD
17KD
8KD

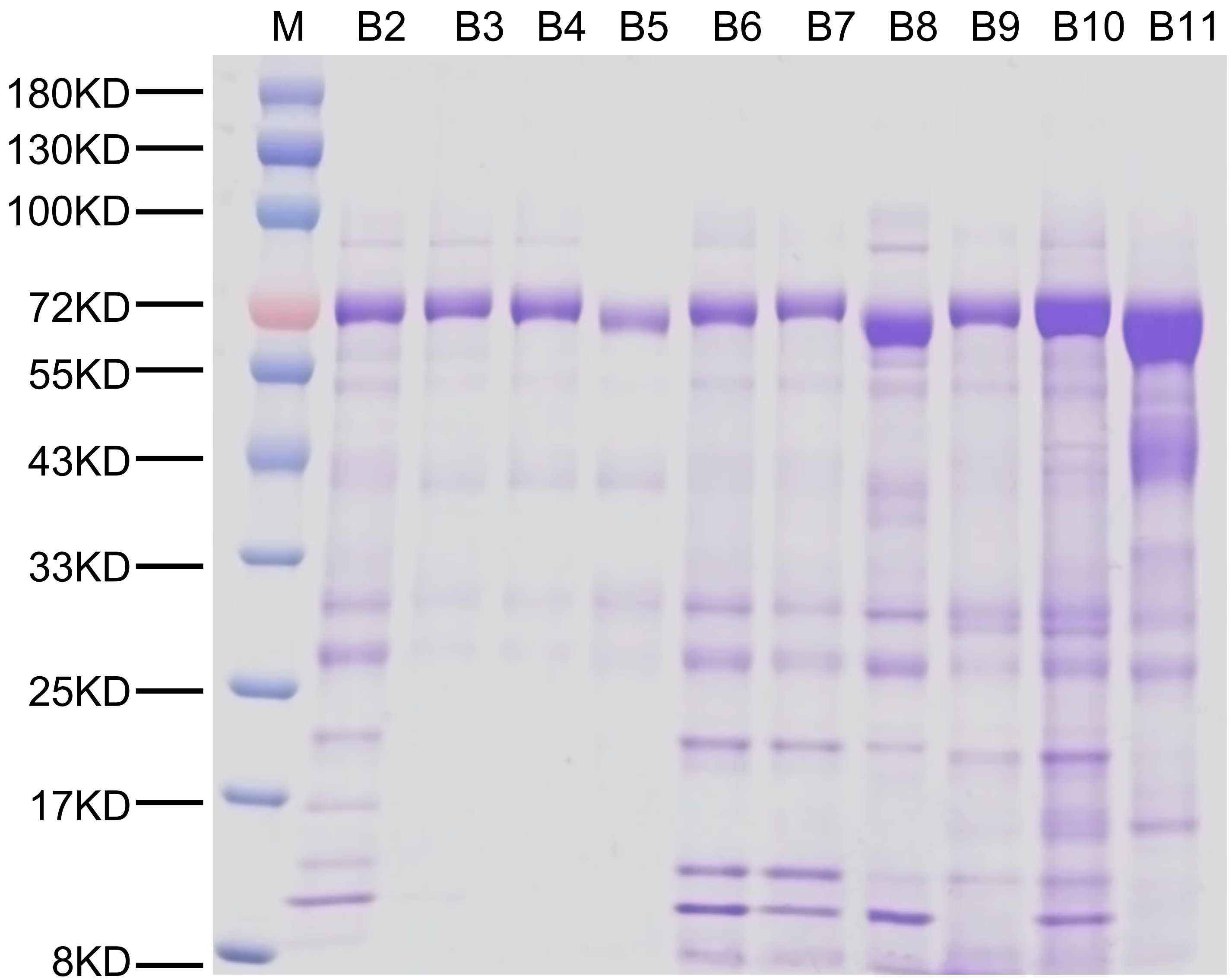

Supplemental Figure 1. SDS-PAGE analysis of total protein in clinical samples. Total protein extracts from the study groups were separated on a 12% resolving gel and visualized by Coomassie Brilliant Blue staining to assess sample integrity and loading consistency. Lane M: Protein molecular weight marker (kDa); Lane A: Sepsis group; Lane B: Septic shock group.
